# Focal therapy in prostate cancer: Development, application and outcomes in the United Kingdom

**DOI:** 10.1002/bco2.70000

**Published:** 2025-02-20

**Authors:** Nadia Rokan, Deepika Reddy

**Affiliations:** ^1^ King's College Hospital NHS Foundation Trust London UK; ^2^ Guy's and St Thomas' NHS Foundation Trust London UK

**Keywords:** cryotherapy, focal therapy, HIFU, IRE, prostate cancer

## Abstract

Prostate cancer is a significant health issue in the United Kingdom, with rising incidence rates prompting the exploration of innovative treatment options. Focal therapy has emerged as a targeted approach that aims to treat localised prostate cancer while minimising damage to surrounding healthy tissue and subsequent adverse side effects. Focal therapy is National Institute for Health and Care Excellence (NICE)‐approved treatment modality for patients with intermediate‐risk localised prostate cancer. This is an evolving field, reflecting the rapidly improved understanding of both the trajectory patients face following a diagnosis of prostate cancer, and how best to apply ablative techniques.

In this narrative review, we evaluate the historical development, current practices, clinical outcome reported in UK‐based studies, and future directions of focal therapy for prostate cancer in the United Kingdom, highlighting its evolution as a viable treatment option.

## 1 INTRODUCTION

Prostate cancer is the most common cancer among men in the United Kingdom, accounting for a substantial portion of cancer diagnoses. The latest reports indicate that one in eight men will be diagnosed with prostate cancer in their lifetime, underscoring the importance of effective treatment strategies. Traditionally, treatments such as a radical prostatectomy and radiotherapy have been the mainstay; however, these methods despite technological advances often come with significant side effects, including urinary incontinence and erectile dysfunction and bowel toxicity. Focal therapy has developed as an alternative, aiming to selectively and successfully ablate cancerous tissue while preserving healthy prostate function.

Cryotherapy dates back to ancient Egyptian times and has developed greatly over the past 200 years with cryosurgery being used for specific, focal destruction of tissues.^1^ The National Institute for Health and Care Excellence (NICE) have published guidelines since 2005 for its use in prostate cancer treatment, under the premise of ensuring continued research and auditing of outcomes and specifically approved for focal treatment in 2012 under the same circumstances.^2^, ^3^ High‐Intensity Focused Ultrasound (HIFU) has been available as a treatment for prostate cancer in the United Kingdom since 2005 with NICE approval within trials or prospective registries granted in 2012 confirmed in 2023, with the caveats of recording all data and publishing results.^4^, ^5^, ^6^ Irreversible electroporation (IRE) for treatment of prostate cancer is the most recent option to have gained NICE approval in 2016, most recently being upgraded from ‘research only’ guidance to ‘special arrangements’ of informed consent, data collection and follow‐up, in 2023.^7^ This paper discusses the evolution, techniques, outcomes and future prospects of focal therapy for prostate cancer in the United Kingdom.

## 2 MECHANISM OF CELL DEATH USING ABLATIVE ENERGIES

Cryotherapy utilises the Joule–Thomson effect enabling freeze‐thaw of tissue. This is created by the interstitial application via cryoprobes of a cryogen agent such as argon gas, followed by the thawing effect of helium gas. The freezing phase requires temperatures of at least −40° C for at least 1 min to induce immediate disruption of the cell membrane bilayer, formation of ice crystals leading to intracellular dehydration due to the osmotic effect and apoptosis induced by mitochondrial damage. Further cell death is induced during the thaw phase, where extracellular ice crystals fuse, resulting in water shifts into the intracellular compartment, amplified by cellular swelling from warmth inducing vascular hyper‐permeability. Repeated freeze cycles result in vasoconstriction and vascular stasis, inducing anoxic and hypoxic cell damage, endothelial damage resulting in platelet aggregation and micro‐thrombi formation and eventual necrosis. Following treatment, leakage of intracellular components results in cryoactivation of anti‐tumour immune response pathways and further cell damage.^8^, ^9^, ^10^, ^11^

HIFU utilises tightly focused ultrasound waves of high intensity (>1260 W/cm^3^), directed specifically at an area of tissue for treatment, typically at most 3.5 cm away from the piezoelectric transducer.^12^ The area of focus is typically 10–12 mm long and 1–2 mm wide, with sequential positioning allowing larger areas to be treated.^13^, ^14^ The ultrasound beam absorption, dependant on the tissue absorption coefficient, results in a rapid temperature rise of between 55 to 90°C, inducing coagulative necrosis. This is followed by target cavitation and subsequent cell destruction. The short bursts of HIFU followed by pausing of ultrasound delivery allows for cooling and minimisation of damage to surrounding tissue whilst maximising damage to targeted tissue. The thermal effects act in conjunction with the mechanistic effect of compression and rarefaction induced by the alternating cycles of ‘power on’ and ‘power off.^15^’ Rarefaction produces microbubbles, which resonate according to pressure changes. Sudden collapse of the microbubbles results in rapid release of energy, a rise in temperature leading to further cell damage and cavitation.

IRE involves a direct current generator and the placement of percutaneous transperineal monopolar electrode needles to specific areas of the prostate. It aims to destroy cancerous cells via a non‐thermal technique, where short electrical impulses of high voltage current are then delivered via the needles, resulting in nanopore formation and an increase in cell membrane permeability.^16^ Such changes lead to ionic shifts and loss of macromolecules, homeostasis disruption and subsequent apoptosis. It was initially deemed the upper limit and avoided when performing reversible electroporation in different treatments, however, was later recognised as a method of inducing specific cell death beyond possible repair, and histopathological analysis has proven its safety to surrounding NVBs, blood vessels, urethra and rectum. Treatment is adjusted by tailoring the number of needles and voltage delivered, with paralysis of surrounding muscles to prevent contractions and disturbed delivery.

## 3 HISTORICAL CONTEXT

Ablative technologies for prostate pathologies have been used since the early 2000s. To address the side effect profile of radical treatments, including whole‐gland ablation, focal ablation was developed.^17^ First used in the early 2000s, focal cryotherapy appeared to be comparable in oncological control over 5 years compared to whole‐gland cryotherapy but demonstrated better preservation of erectile function.^18^ Since the transition to focal ablation in the early 2000s, through large RCTs such as PIVOT, SPCG‐4 and ProtecT, we now have a better understanding of which disease is likely to progress and which is not.^19^, ^20^, ^21^ Notably, the index lesion hypothesis to prostate cancer progression stands at the foundation of the use of focal treatment, with the use of MRI and targeted biopsies enabling accurate identification and localisation of the index lesion.^22^, ^23^, ^24^

## 4 CHANGING TECHNIQUES IN FOCAL THERAPY

Initially developed for the treatment of externally accessible tumours, the use of cryotherapy evolved from open cryoprobe placement to percutaneous placement using TRUS guidance. Transition to minimally invasive, image‐guided techniques enables accurate monitoring of ice ball formation. The rates of rectourethral fistula and urethral structuring rapidly dropped.^25^, ^26^, ^27^ Further evolution from using liquid nitrogen to argon gas enabled accurate and predictable freezing of tissue further safety in the introduction of thawing helium gas providing a safety net of arresting and reversing ice ball formation. The repeated freeze–thaw cycles, typically a minimum of two, are thought to provide a synergistic effect on cell damage within the prostate.^10^ Predictable freeze/thaw and preservation of important structures are confirmed using the application of thermocouples intra‐operatively. Finally, cryoprobe development allowed smaller diameter probes, thus more precise and bespoke treatment delivery and fewer bleeding‐related complications.

HIFU techniques have also evolved over time. Initial development of TRUS‐guided treatment has been adapted to incorporate MRI fusion, allowing for greater accuracy determined by biopsy proven localisation of disease. In field, failures have been associated with lower energy densities; thus, software updates have included visualisation of acoustic energy transmitted and energy density during treatment tissue change monitoring and the ability to increase energy delivered during treatment.^28^, ^29^, ^30^ HIFU technology now allows for tailoring of the focal length, treated areas result in oedema and cavitation, which may dampen further ultrasound propagation. Subsequently, the technique has been adapted to treat the most anterior area first to avoid inadequate energy delivery to targeted tissue. Huber et al. reported the use of ‘double tap’ in which three‐layered blocks to deliver repeated energy over a larger area compared to two overlapping blocks resulted in reduced diagnosis of recurrence following first HIFU treatment.^31^ The current accepted practice to use 4 or 3 cm zones for the first two ablation fields followed by a 3 cm zone. Such protocol increases the area of treatment overlap and minimises skip lesions associated with prostate swelling during HIFU administration. Finally, given the known focal length of most HIFU devices are 4 cm, it is now understood that lesions >3.5 cm from the rectal wall may risk undertreatment to allow for the minimum acceptable treatment margin of 5–8 mm. Thus, patients with more anterior lesions or in larger prostates should be considered for other ablative modalities.

The first human study of IRE in prostate cancer was recorded in 2014 using the ablate and resect method.^32^ This showed successful cancer cells destruction without damage to neighbouring tissue and only mild postoperative haematuria; however, did elicit regional tissue necrosis and inflammatory infiltration. Subsequent following trials have adapted their techniques.

The phase I–II trial implemented the use of four electrodes delivering 90 pulses of 70–100 μs pulse length at 1500 V/cm.^33^ Histology following radical prostatectomy 3 weeks later demonstrated no residual cancer. Since then, electrode numbers, energy delivery, imaging and positioning have all been researched and developed.^34^, ^35^

A prospective study of men treated with IRE for anterior prostate tumours showed significant residual disease at 6 months, and it was determined that mpMRI underestimates treatment volume by up to 9 mm; therefore, the safety margin should be increased up to 10 mm when target planning.^36^

Importantly, the understanding of how the ablative technologies work enables the user to harness the advantages of some techniques whilst avoiding the potential deleterious effects, a concept of ‘a la carte’ treatment as described by Sivaraman et al.^37^ For example, HIFU enables accurate ablation up to 4 cm away from the ultrasound transducer in the rectum, the nature of the ultrasound focussing minimises the heating of tissues more proximal to the focal point thus maintains rectal safety. However, HIFU is greatly impacted by disease more anterior that 4 cm, prostatic calcifications result in reflection of the ultrasound and unpredictable ablation. Interstitial techniques such as IRE and cryotherapy allow for targeting of more anterior lesions that HIFU may not permit. Cryotherapy theoretically can risk rectal toxicity due to ice ball propagation beyond the capsule; thus, in the United Kingdom is rarely used for posterior tumours. For periurethral tumours, IRE has been proven safety, allowing accurate carving of the ablation zone around the urethra but is limited by UK‐based medium‐term outcomes.

## 5 CURRENT FOCAL THERAPY USES

The success of focal therapy largely depends on accurate patient selection. Criteria for suitable candidates typically include low risk with high volume, intermediate and carefully selected high‐risk prostate cancer patients, with localised tumours, often classified as Gleason scores 7 or less.^38^ The overall health status and comorbidities of patients may influence treatment options and their eligibility for focal therapy. Ideal patients for focal therapy are those with significant prostate cancer with a visible lesion on mpMRI and concordant ISUP grade group 2 or 3 cancer on targeted and systematic biopsies. These criteria help to ensure that patients receive the most appropriate treatment tailored to their specific presentations.

Since gaining approvals by NICE, focal therapy has been gaining traction, multiple centres focal therapy provide HIFU, cryotherapy and IRE and incidence of treatment are increasing.^39^

## 6 CLINICAL OUTCOMES IN THE UNITED KINGDOM

Clinical outcomes for focal therapy have been promising, with numerous studies documenting its efficacy. Research based upon patients treated in the United Kingdom indicates that focal therapy can achieve cancer control rates similar to traditional treatments.^40^, ^41^, ^42^, ^43^, ^44^ Propensity‐score‐matched studies demonstrated no significant differences in failure free survival in the medium term (80% following radical treatment, 73% following focal treatment at 6 years and 83% following radical prostatectomy vs. 79% following focal therapy at 8 years).

Patients undergoing focal therapy also reported improved quality of life, with fewer complications related to urinary and sexual function than what is typically reported in cohorts undergoing radical treatments.^45^, ^46^ Patients requiring repeated focal treatments still have considerable preservation of genitourinary function.^47^, ^48^ Patients treated at a tertiary UK centre requiring salvage prostatectomy due to recurrence following focal therapy reported low postoperative morbidity, with only 1/23 patient reporting a Clavien‐Dindo Grade 1 complication.^49^ Further no deterioration in bowel function was reported, and urinary symptoms were preserved or even improved. Despite more recent adoption, IRE demonstrates excellent functional preservation with two thirds of patients treated in the NEAT trial harbouring no clinically significant disease on follow‐up biopsy at 6 months.^50^

Reassuringly, medium‐term outcomes following focal therapy in older patients remain a low morbidity treatment alternative to both radical and systemic treatment.^51^ Over 80% of older patients treated with focal therapy required no further treatment by 5 years. Despite greater failure free survival within the radical treatment group than focal therapy group following propensity‐score‐matched analysis, the lack of long‐term follow‐up may report on the concurrent use of ADT used prior to radical radiotherapy, thus representing an overrating of treatment success.

Long‐term clinical outcomes of the durability of these treatments are still being gathered, and ongoing studies aim to further understand best treatment options with established comprehensive treatment protocols. The PART study, which is now open to multiple NHS sites across the United Kingdom, aims to randomise men to receiving either focal HIFU/IRE or radical prostatectomy.^52^ Such a trial will take years to recruit, additional time will be required for outcomes to mature and provide meaningful analyses.

## 7 REGULATORY AND ECONOMIC CONSIDERATIONS

The NICE plays a crucial role in evaluating the safety and cost‐effectiveness of focal therapy in the United Kingdom. In 2019, the NICE published guidelines recommending HIFU for selected patients, emphasising its potential benefits as a treatment option within the NHS.

Economic evaluations suggest that focal therapy currently provides a cost‐effective alternative to traditional treatments, particularly for low‐to‐intermediate‐risk patients.^53^ Focal therapy provides greater Quality of Life Adjusted Years and incremental net monetary benefit compared to either radical radiotherapy or radical prostatectomy. As healthcare resources become increasingly constrained, the ability to deliver effective treatments with fewer side effects is of paramount importance.

## 8 CHALLENGES AND LIMITATIONS

Although there are many advantages, focal therapy faces several challenges. While short‐to‐medium‐term outcomes are promising, the lack of long‐term data is still needed to ascertain the durability of cancer control and potential late‐onset side effects. Despite consensus statements supporting standardisation of ablative techniques, patient selection and follow‐up protocols, the successful provision of focal therapy continues to be operator dependent.^38^, ^54^, ^55^ Focal therapy requires specialised training and expertise, which may not be uniformly available across all healthcare settings in the United Kingdom at present. The majority of treatment centres are currently in the South of England, and addressing this gap is crucial for broadening access to focal therapy for all eligible prostate cancer patients.

## 9 FUTURE DIRECTIONS

The field of focal therapy continues to evolve with ongoing research and development identifying advanced treatment plans. Technological innovations have incorporated imaging techniques, such as the integration of artificial intelligence in mpMRI analysis, having the potential to improve tumour characterisation and treatment planning. Additionally, the development of new energy delivery systems may enhance the precision and efficacy of existing therapies.^37^

Combination therapies that integrate focal therapy with systemic treatments are being explored, which may enhance overall outcomes for patients with more aggressive disease. Research into synergistic effects between focal therapies and hormonal therapies is ongoing.^56^

Understanding the role of focal therapy in high‐risk patients will be essential for its continued development, and future studies will likely focus on growing patient selection criteria to include those with more advanced disease.

## 10 CONCLUSION

Focal therapy represents a significant advancement in the management of prostate cancer in the United Kingdom. With its ability to provide effective treatments while minimising side effects, it has become a valuable option for selected patients. The ongoing research, technological innovations and regulatory support will be critical in solidifying its role in routine clinical practice. As the understanding of prostate cancer evolves, so too will the strategies to combat it, with focal therapy likely to play a central role in future management paradigms.

## AUTHOR CONTRIBUTIONS

Both authors have contributed equally to the production of this manuscript.

## CONFLICT OF INTEREST STATEMENT

D Reddy has received travel grants from Sonablate Corp. to attend conferences. N Rokan has no conflicts to declare.
